# CoRTE: a web-service for constructing temporal networks from genotype-tissue expression data

**DOI:** 10.1093/bioadv/vbaf272

**Published:** 2025-10-31

**Authors:** Pietro Cinaglia, Mario Cannataro

**Affiliations:** Department of Health Sciences, Magna Graecia University, Catanzaro, 88100, Italy; Data Analytics Research Center, Magna Graecia University, Catanzaro, 88100, Italy; Data Analytics Research Center, Magna Graecia University, Catanzaro, 88100, Italy; Department of Medical and Surgical Sciences, Magna Graecia University, Catanzaro, 88100, Italy

## Abstract

**Motivation:**

A comprehensive and in-depth deciphering of the dynamics concerning gene expressions is essential for understanding intricate biological mechanisms; for instance, the latter can be effectively addressed via network science, and Gene Co-expression Networks (GCNs), specifically. However, a typical GCN is based on a static model, which limits the ability to reflect changes that occur over time. To overcome this issue, we designed an open-source user-friendly web-service for constructing temporal networks from genotype-tissue expression data: *COnstructing Real-world TEmporal networks* (CoRTE).

**Results:**

CoRTE bases the construction of a temporal network on the statistical analysis of the related gene co-expressions across successive age ranges, to define an ordered set of time points. In our experimentation we investigated gene co-expression dynamics across age groups in brain tissues associated with Alzheimer’s Disease, processing curated aging-related data via the proposed web-service. The latter has effectively generated the temporal network consisting of a set of gene pairs that showed statistically significant co-expressions over time. Results demonstrated its capacity to capture time-dependent gene interactions relevant for aging-related disease progression. From a purely applicative point of view, CoRTE may be particularly suitable for exploring aging-related changes, disease development, and other time-dependent biological events.

**Availability and implementation:**

CoRTE is freely available at https://github.com/pietrocinaglia/corte-ws.

## 1 Background

Biological systems consist of highly interconnected units; therefore, network-based models can be effectively suitable for capturing their inherent dynamics. Biological networks are inherently dynamic systems, typically represented through graph-based models in which nodes correspond to biological entities (e.g. genes, proteins, or drugs) and edges denote their interactions or associations. In this context, Gene Co-expression Networks (GCNs) serve as a valuable tool to explore the coordinated transcriptional activity of genes (Cortijo *et al.* 2020). However, conventional GCNs are static by design, limiting their ability to capture the dynamic nature of gene-gene relationships over time, and these are heavily influenced by the specific co-expression measures or correlation methods employed which may obscure time-dependent patterns (Ovens *et al.* 2021). To overcome this limitation, temporal networks, rather than static ones, can be used when temporal dynamics are relevant, offering a powerful extension of the (traditional) static modelling approach (Cinaglia and Cannataro 2022). Formally, a temporal network extends a typical edge in a *contact* (i.e. temporal edge), which is able to represent an interaction that evolves over time. If we wanted to over-summarise, we could say that a contact is the dynamic version of a (traditional) edge that can express the temporal dynamics of an interaction between two nodes of interest. As such, any dynamic system, incorporating temporal information, can be effectively modelled using a temporal network.

In this context, some recent efforts have focused on constructing synthetic Temporal GCNs (TGCNs) from either simulated or real gene expression data. For instance, Li *et al.* (2020) proposed TGCnA, a method for building time-point specific GCNs based on a low-rank plus sparse model. This solution models an ordered sequence of covariance matrices instead of a single one, by producing gene-gene correlation matrices which could serve as the input of GCN analysis. Briefly, it is able to extract the time-invariant latent factors from gene-expression data in order to perform time point specific gene-gene covariance matrix estimation. Therefore, the purpose of TGCnA falls within the scope of our solution, but cannot be assimilated to it in terms of objective or for a possible performance comparison. Specht and Li (2017) proposed LEAP, an algorithm for the computation of gene co-expressions that allows evaluating possible lags over time. It is able to construct GCN for single-cell RNA-sequencing data across experiments using a pseudo-time ordering; by definition, the latter is assigned based on changes in the cell transcriptome for inferring dynamics in biological processes. Similarly to the previous one, this study, although relevant, also deviates from our primary objective.

Considering this notable gap in the availability of software tools capable of integrating temporal information in network models, and more specifically for inferring TGCNs, we designed an open-source user-friendly web-service for constructing TGCNs from real genotype-tissue expression data: *COnstructing Real-world TEmporal networks* (CoRTE). The choice to operate only on real-world and curated data comes from the assumption that although the synthetic one is useful for testing and benchmarking, the former provides more biologically relevant insights, especially when drawn from diverse tissue types and developmental stages (Dai *et al.* 2025).

CoRTE constructs TGCNs in which the nodes represent the genes, and the contacts (handled as a set of edges for each time point of interest) indicate statistically significant co-expressions over time. GCNs capture tissue-specific transcriptional and splicing regulation, which underscores the importance of enabling users to select specific tissues of interest. This feature allows for the generation of more biologically meaningful temporal networks, enhancing the interpretability and relevance of observed co-expression patterns.

## 2 Materials and methods

CoRTE provides a systematic pipeline for constructing temporal networks from genotype-tissue expression data, capturing time-dependent biological interactions, particularly gene expression variations across tissues and age groups.

It adopts a snapshot-based representation, consisting of a set of static networks representing the temporal network in a specific time point (i.e. snapshot). We denote the temporal network by $TN=[S_{1},S_{2},\ldots ,S_{n}]$, where $S_{i}=(V,E_{i})$ represents the *i*-th snapshot observed at a specific time point ($t_{i}$), with 0≤i≤n, and *n* indicating the total number of time points; note that the node set *V* consists of all nodes, and it is assumed to be immutable.

The tool integrates data from the Genotype-Tissue Expression (GTEx) project (GTEx Consortium 2013), thereby enabling construction of time-evolving networks in which nodes represent genes and temporal edges (i.e. contacts) reflect statistically significant co-expression relationships. GTEx public data are retrieved by using the official programmatic access made available via an Application Programming Interface (API, https://gtexportal.org/home/apiPage); specifically, we exploited the following endpoints:

- *reference/gene*: this endpoint provides reference gene metadata, including Gene Identifier (Id), symbol, type, and a functional description.
- *expression/geneExpression*: this endpoint provides expression profiles, necessary for determining co-expression relationships between genes. The gene expression data are normalized, and these concern a specified set of genes defined as an input parameter.

The first is used for integrating the information entered by the user, in order to match the gene aliases with the respective unique Ids used by GTEx (i.e. GENCODE Id). The second is used for evaluating the presence and strength of gene-gene associations, in terms of statistically significant co-expressions.

CoRTE requires the following user-defined parameters: (i) a list of genes of interest (mandatory), (ii) a list of tissues to be used for filtering expression data (optional; default: all tissues are included), and (iii) a significance threshold for correlation analysis (optional; default: 0.05).

Temporal information, derived from discrete age groups, allows for analysing gene expression dynamics across age-defined time points. The age groups, in which GTEx data has been organized, are reported as follows: [20–29], [30–39], [40–49], [50–59], [60–69], and [70–79]; the unit is the year. CoRTE uses each age group to model the corresponding snapshot (i.e. time point) within the temporal network. Accordingly, expression data is filtered and grouped by age, enabling time-resolved co-expression analysis. Additionally, users may choose to also indicate a set of tissues to circumscribe the analysis, in order to contextualize the co-expression of the indicated genes according to one’s own purposes.

The determination of the existence of an edge at a specific time point is performed on the basis of the statistical correlations between the pairs of genes (i.e. nodes) for each age group. Specifically, CoRTE uses Pearson’s correlation coefficient for quantifying the degree of linear association between gene pairs, while a *P*-value threshold is employed for hypothesis testing (default: *P* < 0.05). We based the computation of correlations on this measure in that it is widely used in determining relationships in network science, and more specifically, its effectiveness is well-discussed in literature for GCN analysis (Hou *et al.* 2022). According to Ovens *et al.* (2021), Pearson’s correlation coefficient measures the extent of a linear relationship between variables *x* and *y* and is a preferred and standard way of calculating GCN edge weights. It is able to process a significance filtering which removes spurious associations and enhances the biological interpretability of the resulting network. Notably, this threshold is user-configurable to accommodate different analytical needs.

Summarizing, the set of statistically significant correlations identified for each time point determines the existence of corresponding temporal edges in the related snapshot. Furthermore, the use of GTEx API ensures that data always remains up-to-date and in line with the official source that curated it. It’s important to note that our method performs data processing by parallelizing operations based on time points. This allows time points to be processed independently and in parallel, ensuring optimized performance and runtimes.

Below we illustrate our method by reporting what has already been described in the form of pseudocode, and by exploring into the main details.

Algorithm 1 is a non-exhaustive pseudocode focused on the main procedures which constitute the workflow performed by CoRTE for constructing a temporal network in snapshot-based representation, as discussed.

Algorithm 1 Construction of a Temporal Gene Co-expression Network (TGCN) by CoRTE.

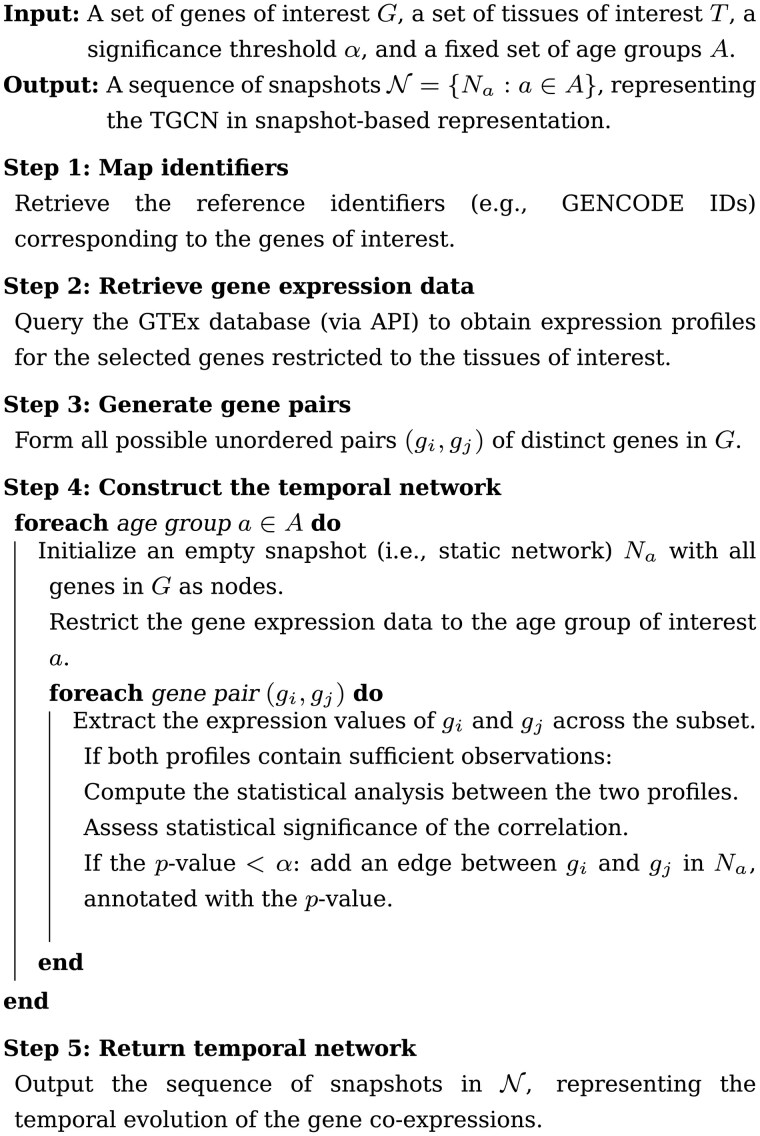

First, all input genes are harmonized to a common identifier space (*Step 1*). Next, expression profiles are retrieved from GTEx, restricted to tissues of interest (*Step 2*). From these data, all possible gene pairs are generated (*Step 3*). For each age group (i.e. time point), a snapshot (i.e. static network) is constructed by statistically analysing the correlation between the expression of each pair of genes (*Step 4*). Specifically, contacts are defined only when the correlation is statistically significant. The output is a TGCN, modelled as a sequence of snapshots that describe the temporal evolution of the gene co-expressions.

Formally, let $G=\{g_{1},g_{2},\ldots ,g_{n}\}$ denotes the set of genes of interest, and *T* the set of tissues under consideration. The set of age groups $A=\{a_{1},a_{2},\ldots ,a_{m}\}$ can be assumed as constant. In *Step 1*, each gene g∈G is mapped to a standardized identifier (i.e. GENCODE IDs), useful to retrieve the expression profiles from GTEx. In *Step 2*, a query processing restricted to the tissues of interest *T* is performed (if this parameter is not defined, then the method will consider all tissues as being of interest). This step yields the expression matrix *X*; it can be denoted as:


X(g,t,a) for g∈G, t∈T, a∈A,

where X(g,t,a) is the multiset of observed expression values for gene *g* in tissue *t* and age group *a*.

*Step 3* produces a set consisting of all unordered gene pairs (*P*), that is used for evaluating the co-expressions between the gene pairs. Briefly, *P* is the set of candidate nodes in the resulting TGCN; let us denote *P* by the following representation:


$$P=\{\{g_{i},g_{j}\}:g_{i},g_{j}\in G,\;i<j\}.$$

In *Step 4*, the method performs the statistical significance analysis (f-stats), for each age group a∈A (i.e. time point) and each pair $\{g_{i},g_{j}\}\in P$, in order to calculate the related statistical significance *p* (or *P*-value):


$$p_{ij}(a)=f-\text{stats}\left(\left\{g_{i},g_{j}\}(a\right)\right).$$

The snapshot $N_{a}=(V,E_{a}),\;V=G$ is built based on the edge set $E_{a}$ given by significant co-expressions between the pair of genes belonging to *P*. Let us denote $E_{a}$ as follows:


$$E_{a}=\left\{\{g_{i},g_{j}\}\in P\;\;:\;\;p_{ij}(a)<\alpha \right\},$$

with α the significance threshold (default 0.05).

The resulting TGCN constructed by CoRTE can be denoted as follows:


$$\text{TGCN}=\{N_{a}:a\in A\}$$

Note that the full and well-structured source code of CoRTE is available in the official repository (see *Data availability*).

### 2.1 Implementation

CoRTE has been implemented in Python, as a web service. It leverages a combination of scientific computing, data processing, and network analysis libraries to construct and analyse TGCNs. It integrates multiple well-established libraries to ensure efficient data retrieval, processing, and visualization.

The Pandas library (https://pandas.pydata.org) was employed for efficient data manipulation, filtering, and transformation of gene expression datasets. Its robust data structures facilitated the preprocessing steps necessary for constructing temporal networks.

To support numerical computations, particularly those related to correlation matrix generation and statistical analysis, we utilized both NumPy (https://numpy.org) and SciPy (https://scipy.org), respectively. In detail, SciPy was used to compute Pearson’s correlation coefficients between the expression profiles of each gene pair (i.e. network nodes), as well as to perform hypothesis testing for statistical significance.

Network modelling was carried out using the NetworkX library (https://networkx.org). However, since NetworkX does not natively support temporal networks, we adopted a modular approach by representing the temporal topology as a list of graph objects, with each one corresponding to a specific time point; in accordance with snapshot-based representation. This design circumvents the limitations of the library while enabling a time-resolved representation of gene-gene associations.

The computation has been parallelized for optimizing performance in terms of runtime; specifically, we used Joblib (https://joblib.readthedocs.io) for handling multiprocessing.

Additionally, FastAPI (https://fastapi.tiangolo.com) was used to expose the service’s features, as well as to manage both requests and data exchange. It is a high-performance web framework for building HTTP-based service API.

Network visualization plays an important role in enhancing readability of temporal network evolution, providing an intuitive way to understand dynamic changes across different time points. If the resulting temporal networks are to be used within another Python algorithm, we recommend employing Matplotlib (https://matplotlib.org), which was also applied during our testing with satisfactory results. However, data produced by CoRTE is also supported by well-known visualization tools such as Cytoscape (https://cytoscape.org), as it is available both as an edge list and an adjacency list. For illustrative purposes only, we report below a snippet of code for plotting a temporal network exported in snapshot-based representation by using CoRTE:

’’’

temporal_network is a list of edge lists (nested lists)

concerning each timepoint (tp) of interests

’’’

def plot(temporal_network: list):

 for i, tp in enumerate(temporal_network):

   plt.figure()

  plt.title(f”Timepoint {i} - {self.AGES[i]}”)

   nx.draw(tp, with_labels=True)

   plt.show()

Alternatively, edge lists (with columns: source, target, pvalue) are compatible with Cytoscape for interactive network visualization and analysis (see Figure 2 as example). These output formats ensure reproducibility and ease of integration with major network visualization platforms.

**Figure 1.**
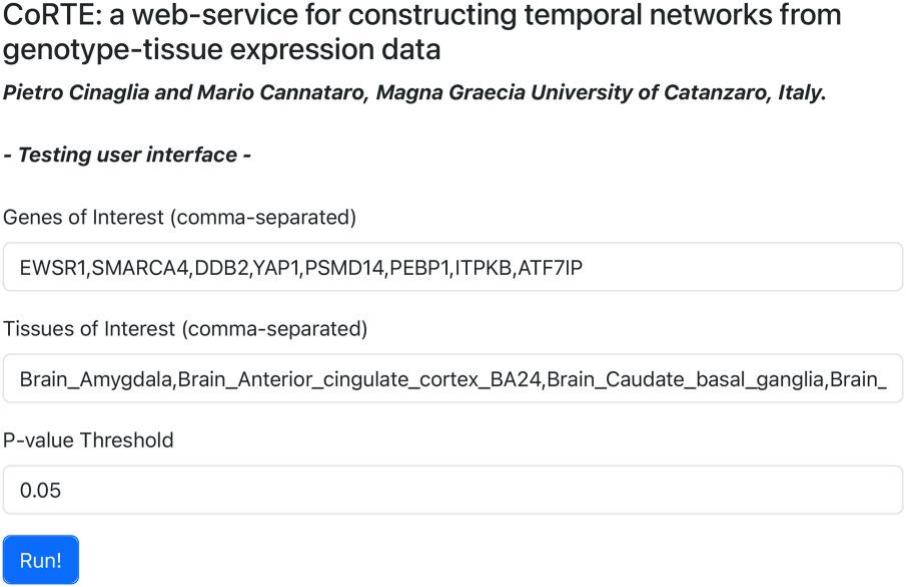
The figure shows a prototype interface to allow the user to easily set parameters such as genes of interest, tissues of interest, and significance thresholds for constructing temporal gene co-expression networks. Designed to improve user experience, it supports experimentation and testing by providing an intuitive form-driven workflow. It facilitates interactive selection and submission of query parameters, enabling efficient exploration of gene expression dynamics across tissues and age groups. Furthermore, it could be crucial to make CoRTE even more accessible and user-friendly for researchers without extensive computer science skills.

For demonstration and testing purposes, we developed a web interface for our service, enabling users to set the parameters through a user-friendly form, in order to also improve the user experience in experimentation and testing operations. It was implemented using HyperText Markup Language 5 (HTML5) and integrated into our solution as a Jinja template (https://jinja.palletsprojects.com); Figure 1 shows its prototype implementation.

## 3 Experimentation

In this section, we discuss a case study to illustrate the application of our solution and assess its effectiveness. It has been focused on aging in relation to the progression of Alzheimer’s Disease (AD). AD has been chosen because it represents both the most common neurodegenerative disorder associated with aging and the leading cause of dementia. For demonstration purposes, we applied CoRTE to construct a temporal network using aging-related genes that Liu *et al.* (2024) identified and validated as novel diagnostic biomarkers for AD: EWSR1, SMARCA4, DDB2, YAP1, PSMD14, PEBP1, ITPKB, and ATF7IP. Data analysis was restricted to brain tissues with high reported gene expression levels in GTEx, specifically: Amygdala, Anterior cingulate cortex (BA24), Caudate (basal ganglia), Cerebellar Hemisphere, Cerebellum, Cortex, Frontal Cortex (BA9), Hippocampus, Hypothalamus, Nucleus accumbens (basal ganglia), Putamen (basal ganglia), Spinal cord (cervical C-1), and Substantia nigra.

**Figure 2.**
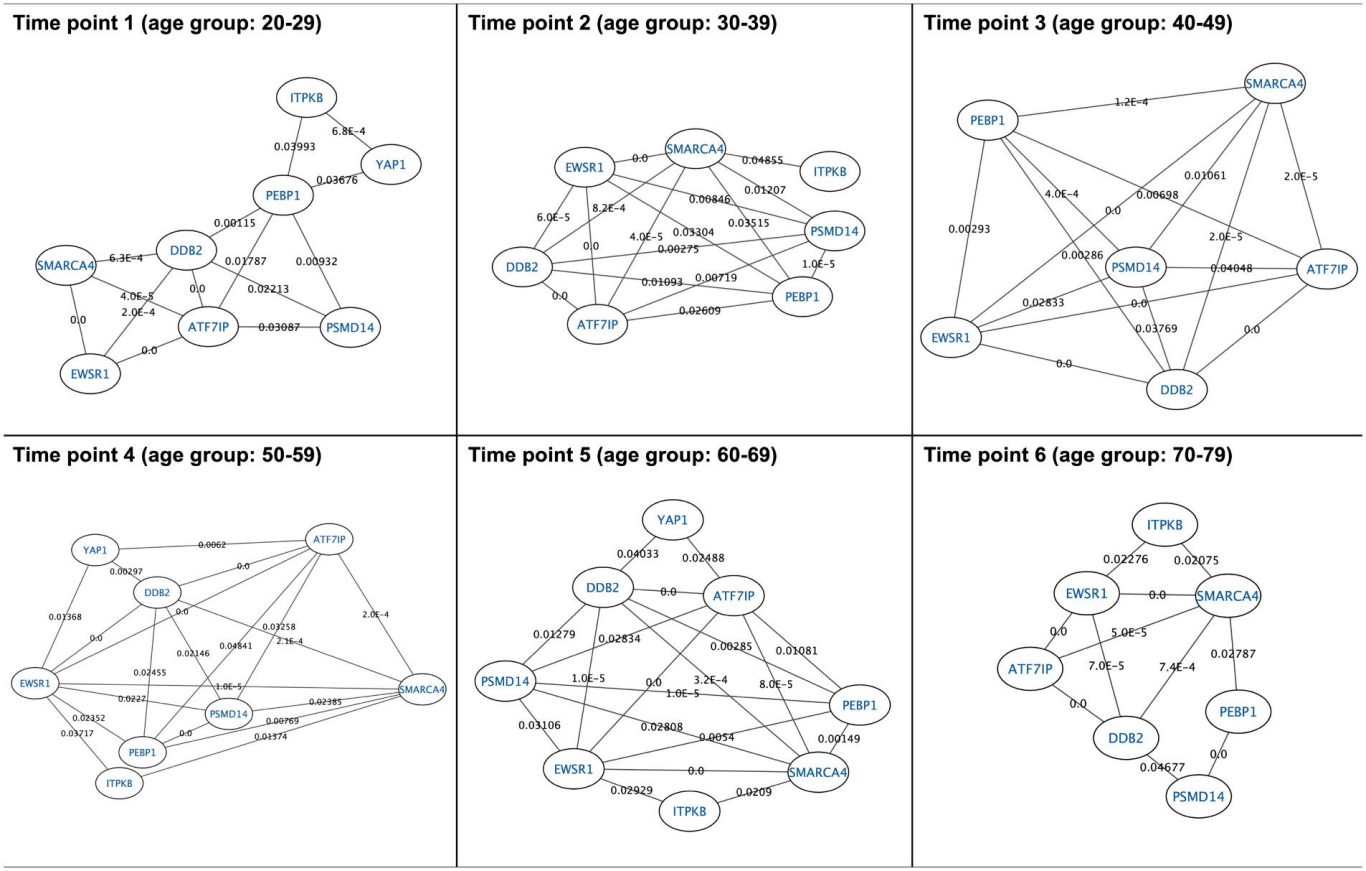
This figure shows the temporal network modelled by CoRTE in our experimentation. It concerns the dynamic evolution of gene-gene associations for aging-related genes in brain tissues. Nodes represent genes, and temporal edges indicate statistically significant co-expression relationships identified within each discrete age group (20–29, 30–39, …, 70–79 years). Edges existing at specific time points are annotated by their *P*-values, a value of 0.0 indicates a *P*-value < 1*e*^−6^. The snapshot-based network model highlights temporal changes in gene connectivity, offering insight into gene regulation dynamics associated with aging and Alzheimer’s disease progression. Note that the plots have been generated by using Cytoscape, specifically, we imported the exported snapshots as a set of edge lists (one for each time point).

As outlined in Section Materials and Methods, CoRTE determines the statistical significance of gene-pair relationships based on expression levels across age groups (i.e. time points). This analysis identifies which temporal edges should be established between nodes (genes) over time. The temporal network was then constructed using this statistical framework, following a snapshot-based approach. Accordingly, CoRTE generated a snapshot for each time point, modelling the overall temporal network. The resulting temporal network is presented in Figure 2 and consists of all significant interactions for which an edge was created within the corresponding time point. This figure consists of plots generated using Cytoscape (see Section Implementation), specifically, we imported the exported snapshots as a set of edge lists (one for each time point). Alternatively, you could also use the TimeNexus plugin (freely available in the *Cytoscape App Store* at https://apps.cytoscape.org/apps/timenexus) to effectively manage the temporal network in a single view. It combines node and edge tables, carrying information on interacting nodes, as well as the gene expression related to each node over time.

## 4 Conclusions

In this paper, we presented CoRTE, a web-service for constructing real-world biological temporal networks from genotype-tissue expression data. It empowers researchers to investigate the temporal dynamics of gene co-expression with fine granularity by leveraging a biologically curated dataset. Its reproducible and scalable design enables the study of aging-related gene regulation and facilitates the discovery of molecular mechanisms underlying development, aging, and disease progression.

### 4.1 Limitations and future works

Our method provides valuable insights but is constrained by several factors. The main limitation concerns the data, which is unevenly distributed across tissues and age groups, limiting robustness in under-represented subsets. This issue could lead to biases in the constructed temporal networks, potentially compromising the robustness and biological interpretability of inferred contacts in under-represented subsets. To mitigate such biases, future updates will incorporate statistical normalization and resampling approaches, including stratified sampling and sample weighting, to balance the representation of age and tissue groups prior to network inference. Additionally, sensitivity analyses may be performed to assess the stability of network topology when confronted with variable sample sizes. Although the proposed solution is currently dependent on GTEx data, it processes gene expression data using standardized data structures, which allows seamless adaptation to alternative data sources after appropriate data harmonization, as well as its modular architecture enables future integration of additional transcriptomic datasets. The reliance on pairwise Pearson correlation captures only linear relationships, overlooking non-linear and higher-order interactions. Based on these assumptions, future works will focus on extending statistical processing by supporting other correlation methods, by also including a set of data analysis features. For instance, future updates will include alternative network inference methods, such as mutual information, partial correlation, and distance correlation metrics. Modular pipeline expansion will allow users to select the preferred inference strategy, enabling more comprehensive exploration of complex transcriptomic interactions. Further, integration of causality-aware or time-lagged methods is under evaluation to enhance detection of dynamic regulatory relationships. Finally, we are considering releasing our solution as a stand-alone module integrable in our in-house pipeline engine [FLENP (Cinaglia 2024)], so that TGCN construction can be handled as a step within more elaborate, yet automated, workflows. The modular pipeline expansion will allow users to select the preferred inference strategy, enabling more comprehensive exploration of complex transcriptomic interactions.

## Data Availability

CoRTE’s source code and additional materials are available on GitHub at https://github.com/pietrocinaglia/corte-ws.
